# Relentless placoid chorioretinitis: A review of four cases in pediatric and young adult patients with a discussion of therapeutic strategies

**DOI:** 10.3389/fped.2023.885230

**Published:** 2023-03-27

**Authors:** Haniah A. Zaheer, Jamie Odden, Meghal Gagrani, Fatma Zaguia, Careen Lowder, Andreea Coca, Margalit E. Rosenkranz, Preeti Patil-Chhablani, Raphaelle Ores, Francois Boussion, Chad Indermill, José-Alain Sahel, Ken Nischal, Debra A. Goldstein, Marie-Helene Errera

**Affiliations:** ^1^ Department of Ophthalmology, Children Hospital Pittsburgh, University of Pittsburgh School of Medicine, Pittsburgh, PA, United States; ^2^ Associated Retina Consultants, Phoenix, AZ, United States; ^3^ Department of Ophthalmology, Northwestern University Feinberg School of Medicine, Chicago, IL, United States; ^4^ Cleveland Clinic Cole Eye Institute, Cleveland, OH, United States; ^5^ Department of Rheumatology, University of Pittsburgh Medical Center, Pittsburgh, PA, United States; ^6^ Department of Rheumatology, Children Hospital Pittsburgh, University of Pittsburgh School of Medicine, Pittsburgh, PA, United States; ^7^ CISSSO ou Centre Intégré Services Sociaux de l’Outaouais, Gatineau, QC, Canada; ^8^ Centre Hospitalier National des Quinze-Vingts, Paris, France; ^9^ Department of Ophthalmology, Centre Hospitalier National des Quinze-Vingts; Sorbonne Universites, Paris, France

**Keywords:** relentless placoid chorioretinitis, uveitis, adalimumab, infliximab, interferon-alpha-2a, tocilizumab

## Abstract

**Introduction:**

Relentless placoid chorioretinitis (RPC) is a rare, bilateral disease of the retinal pigment epithelium. The clinical course is prolonged and relapsing. No standard treatment has been established to date. The purpose of this case series is to report four cases of RPC in pediatric and young adult patients in which varying treatments were used, comparing them to previously published cases.

**Methods:**

A literature review was conducted to investigate currently published presentations and treatment options for RPC. A multicenter retrospective chart review was also performed on four consecutive patients. These patients were diagnosed with RPC because of new chorioretinitis lesions continuing to appear without or despite therapy for 5–36 months (2 patients), with a clinical course prolonged and relapsing, or because of the atypical location of the multiple lesions (>50) extending from the posterior pole to the equator and mid-peripheral retina (all four patients), which were not consistent with other entities like acute posterior multifocal placoid pigment epitheliopathy and serpiginous choroiditis.

**Results:**

All four cases of RPC received oral or IV steroids acutely, and three of these patients were transitioned to a steroid-sparing agent and biologic therapy: anti-TNF alpha or anti-IL-6. Quiescence of the chorioretinitis lesions was obtained after 7 months, 1 month, and 36 months; however, the latter had issues with treatment adherence. Mycophenolate mofetil was insufficient to control the disease in one patient, but tocilizumab and infliximab thereafter were effective after cessation of adalimumab due to side effects. Adalimumab when started the first month after the presentation was effective in controlling the disease in one patient. After the failure of interferon-alpha-2a, one patient displayed long-term control with infliximab. One patient did not require a steroid-sparing agent after oral prednisone taper as there was no evidence of progression or recurrence.

**Conclusion:**

This case series adds to the current knowledge regarding potential treatments for RPC, specifically the use of anti-TNF-alpha treatment and anti-IL-6 tocilizumab. In this case study, relapses of RPC were found among patients on mycophenolate mofetil and interferon-alpha-2a, and one case did not relapse on oral steroids without a steroid-sparing agent. Our findings suggest that adalimumab, infliximab, and tocilizumab may be useful medications to obtain quiescence of RPC.

## Introduction

Relentless placoid chorioretinitis (RPC) is a rare, bilateral disease of the retinal pigment epithelium (RPE) and choroid, first described by Jones et al. (1). Due to the similarities of acute lesions with serpiginous choroiditis (SC) and acute posterior multifocal placoid pigment epitheliopathy (APMPPE), the diagnosis of RPC is often delayed due to disease overlap. The distinction of RPC consists of its atypical time course and retinal distribution. RPC is characterized by >50 bilateral chorioretinal lesions with involvement anterior and posterior to the equator. This is in contrast to SC, where, usually, only one eye contains active lesions at a time, consisting of subretinal infiltrates that spread centrifugally from the peripapillary region and result in a typical geographic scar (2). Similar to SC, however, the clinical course of RPC is prolonged and relapsing, with new lesions continuing to appear for 5–24 months without therapy (3). Patients may show recurrences for months to years after onset. Multimodal retinal imaging suggests that there is a common pathophysiology with APMPPE and SC, although the number of lesions (>50) and location suggest RPC (3).

Early in disease progression, RPC appears similar to APMPPE and SC on fundus autofluorescence (FAF) in that the lesions are hypoautofluorescent with hyperautofluorescent edges. In RPC, fluorescein angiography (FA) studies reveal hypofluorescence in the early phases of the angiogram. Later phases show staining of the lesions. Indocyanine green angiography (ICG) shows hypofluorescence in the areas corresponding to the clinical lesions that persist into the late phases. The hypoﬂuorescence of RPC, like in APMPPE in the early phase of FA and throughout the ICGA sequence, suggests multifocal choroidal hypoperfusion. Both FA and ICG for RPC show a similarity to those for APMPPE and SC (3, 4). This suggests that it might be within the spectrum of inflammatory disease with primary involvement of the level of the choriocapillaris and secondary RPE damage as seen in APMPPE (5). Additionally, active lesions typically show focal areas of hyper-reflectivity developed in the outer retina, primarily localizing to the outer nuclear layer (ONL) but also being seen at the level of the outer plexiform layer on spectral-domain optical coherence tomography (SD-OCT). In lesions that fail to completely resolve, persistent disruption and/or irregularity of the RPE and associated retinal thinning are seen. An OCT-angiography (OCT-A) study has shown that the inner choroid is the primary site of disease pathogenesis in RPC, as well as in APMPPE (6).

No specific underlying etiology has been identified for RPC thus far; however, there may be infectious or autoimmune causes that lead to its presentation. Other systemic illnesses have been suggested to be associated with RPC, such as thyroiditis and cerebral vasculitis (7, 8). Viral or flu-like illnesses have been reported in approximately 33% of patients prior to ocular symptoms of APMPPE (9). During the COVID-19 pandemic, cases of choroiditis, APMPPE, SC, and RPC have been reported in patients infected with COVID-19, which may further support the theory that viral infections may play a role in triggering chorioretinal inflammation in susceptible individuals (10–14).

Multiple cases of RPC have been published in the literature; however, the shape of chorioretinal lesions, clinical course, and prognosis have varied. Early diagnosis is pivotal to ensure intervention before the further progression of the disease. However, no standard treatment for RPC exists; immunosuppressive drugs such as prednisolone, cyclosporine, intravitreal triamcinolone, adalimumab, infliximab, and mycophenolate mofetil (MMF) have been used to treat RPC (1, 8, 15–20). Other studies have also worked at using a combination of these medications (i.e., prednisolone/cyclosporine and adalimumab/infliximab) (1, 8, 15, 16, 19, 20).

The purpose of this paper is to compare the treatment outcomes in four cases of RPC, the largest series in pediatrics and young adults, to previously reported cases of RPC. Specifically, this paper discusses the efficacy of currently published treatments of RPC and the successful use of an anti-IL-6 agent (i.e., tocilizumab) for the first time, to our knowledge, to control the disease.

## Methods

**Figure 1:**
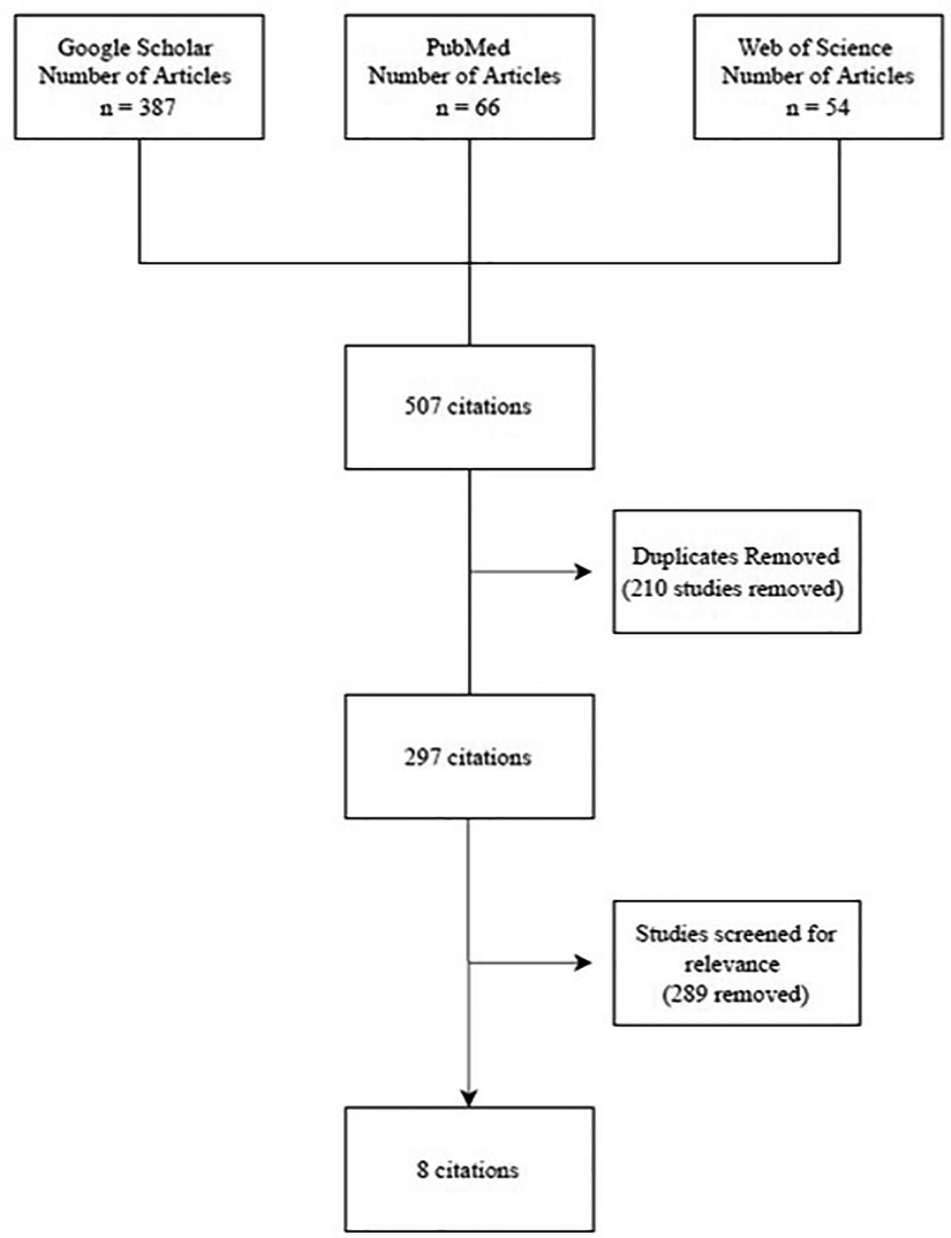
PRISMA flow diagram.

A literature search and subsequent screening of articles were conducted in December 2021 by one author (HAZ). Keywords relating to RPC were used to search the following electronic databases in the given order: Google Scholar, PubMed, and Web of Science. Keywords included *relentless placoid chorioretinitis*, *ampiginous choroiditis*, *relentless placoid chorioretinitis and treatment*, and *ampiginous choroiditis and treatment.* The retrieved articles were initially screened by title, and articles with the relevant titles were then screened by abstract using predefined inclusion and exclusion criteria. Inclusion criteria are as follows: (1) the paper must be in English and (2) the paper must discuss the presentation and management of RPC. Exclusion criteria are as follows: (1) the paper concerned patients only with other white dot syndromes, such as SC or APMPPE; (2) the paper did not clearly diagnose the patient with RPC; and (3) the citations were from gray literature. The full article was screened in cases where the relevance was unclear from the abstract. Relevant articles were ultimately compiled into a database, and duplicates were removed (Figure 1).

For the cases presented in this article, electronic medical records for relentless placoid chorioretinitis were reviewed for the initial presentation, imaging, laboratory studies and results, follow-up visit presentations, and medical management.

### Case 1: RPC

**Figure 2:**
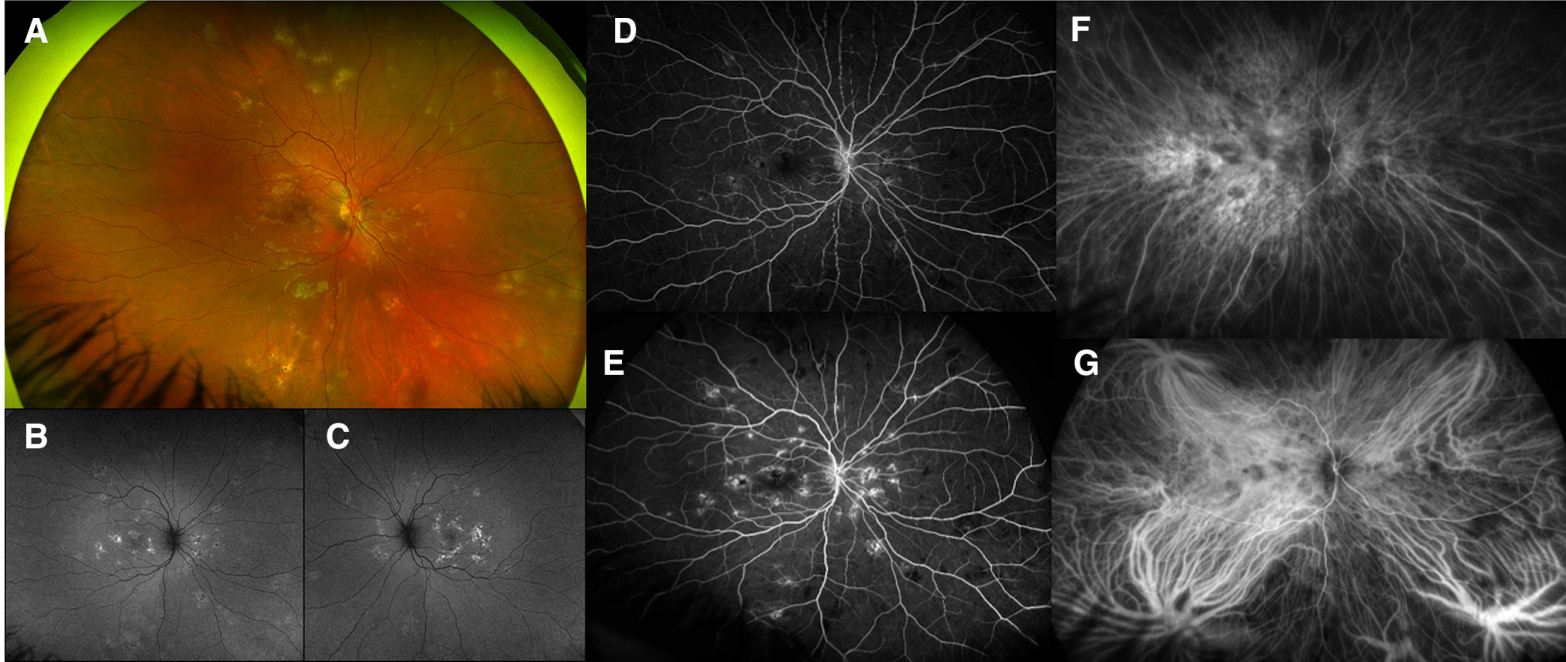
Patient 1. (**A**) Right eye revealed yellow-white placoid lesions in the posterior pole and extending into the mid-periphery. Some appeared gray and healed, and others appeared more white and active (superior nasally). Left eye (not shown) looked similar to the right eye. (**B,C**) Autofluorescence revealed bilateral hypofluorescent lesions with hyperfluorescent edges. (**D**) Early fluorescein angiography (FA) showed early hypofluorescence of lesions. (**E**) FA showed late staining of the lesions. (**F**) ICG showed hypofluorescence of the lesions. (**G**) Late ICG showed continued hypofluorescence of the lesions.

A 17-year-old girl with two previous episodes of chorioretinitis in the left eye in the last 12 months, seen elsewhere, presented with 1 month history of bilateral scotomas and photopsias, headache, and back pain. Her familial history was remarkable for only multiple sclerosis. She had been previously started on 1 mg/kg/day of oral prednisone with slow tapering and is currently on 20 mg daily. Her vision was 20/25 in the right eye and 20/60 (20/30 pinhole) in the left eye. A slit lamp exam showed 2 + cells in the anterior chamber in the right eye and no vitritis. Her fundus exam revealed yellow-white and gray placoid lesions in the posterior pole extending into the mid-periphery in both eyes (Figure 2A). Fundus autofluorescence (FAF) showed hypofluorescent lesions with hyperfluorescent edges (Figures 2B,C). SD-OCT revealed ellipsoid (EZ) layer loss in the parafovea of both eyes. On FA, both eyes demonstrated early hypofluorescence and late staining of the lesions (Figures 2D,E). On ICG, the lesions appeared hypofluorescent in both eyes in early and late phases (Figures 2F,G).

**Figure 3:**
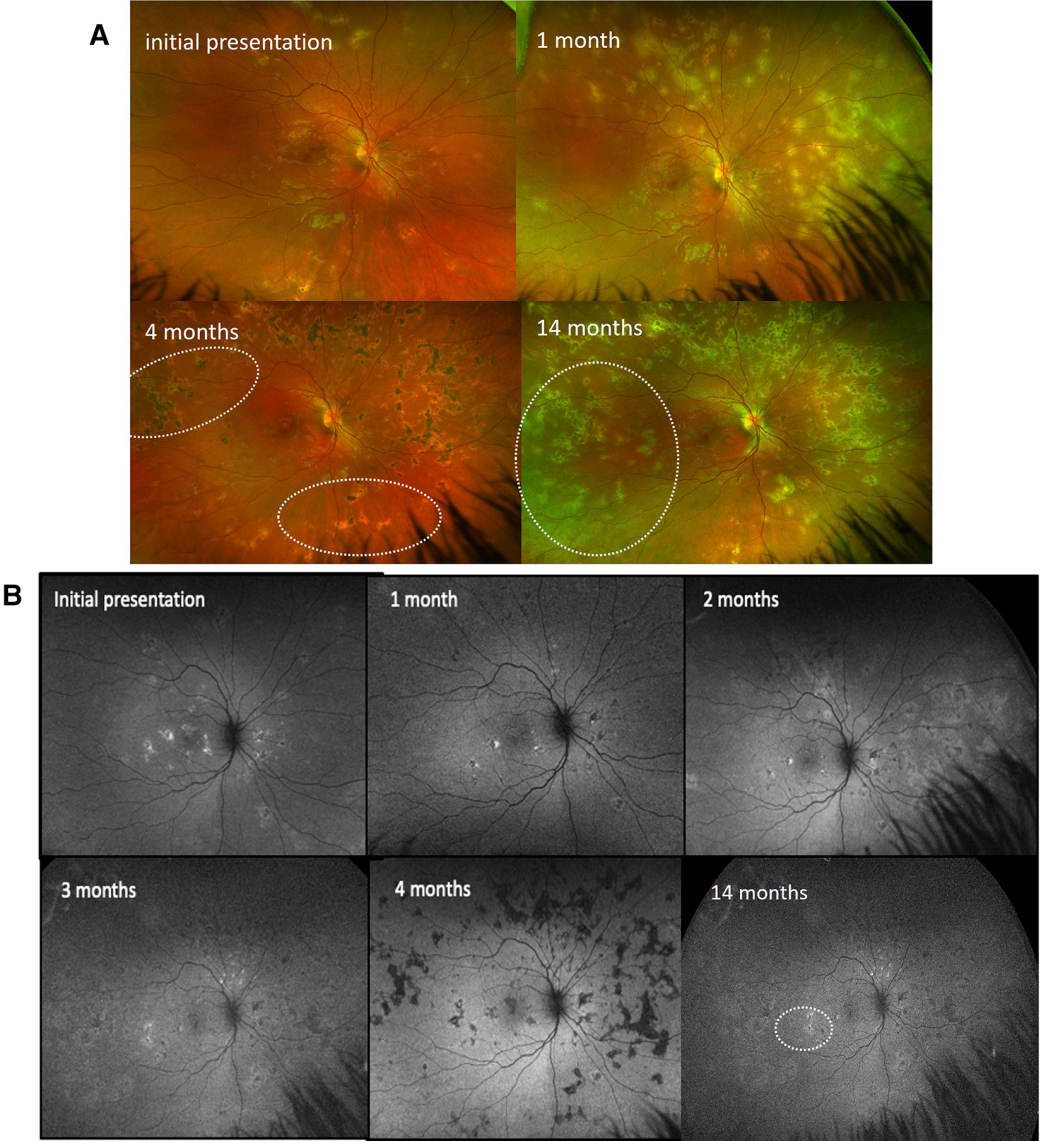
Patient 1. (**A**) Ultrawide-field fundus color imaging over time. Right eye only. The left eye demonstrated similar findings. The placoid lesions increased in quantity over time from initial presentation, 1 month, and 4 months to 14 months after presentation. (**B**) Fundus autofluorescent studies over time. Right eye only. The left eye demonstrated similar findings. The hypo/hyperautofluorescent lesions increased in quantity over time from initial presentation, 1 month, 2 months, 3 months, and 4 months to 14 months after presentation.

The uveitis work-up was negative (details are in Table 1). The patient was started on oral mycophenolate mofetil (MMF) 250 mg twice a day, and oral prednisone was decreased to 0.75 mg/kg/day with gradual tapering (Table 2). At 1, 2, 3, and 4 months after the presentation, more yellow-white and gray placoid lesions were noted on fundus color pictures (Figure 3A) that appeared hypo/hyperautofluorescent on FAF in both eyes (Figure 3B). No clinical efficacy of the increased dose of MMF to 1,000 mg twice a day and oral prednisone (1 mg/kg/day) was noted; therefore, subcutaneous injections (SQ) of adalimumab were initiated. Due to cutaneous side effects, she was switched to tocilizumab at 7 months after the presentation, which led to quiescence of the fundus lesions noted soon thereafter. Eleven months later, tocilizumab was discontinued due to a minor surgical procedure. Fourteen months later, a new symptomatic choroiditis lesion was noted at the macula (Figure 3A) that showed hyperautofluorescence (Figure 3B) with outer retinal hyperreflectivity on SD-OCT. IV infliximab (5 mg/kg) and oral methotrexate (10 mg weekly) were started. Good efficacy of infliximab was noted clinically at the last follow-up, 12 months later, and in terms of visual outcomes. Her final vision was 20/15 in both eyes with bilateral subjective scotoma.

**Table 1:** Summary of four cases of relentless placoid chorioretinitis.

Case #	Eye affected (OD, OS, OU)	Symptoms	Age, sex	Uveitis work-up	Clinical findings	Fundus exam	FA	ICG	FAF	SD-OCT	Timing of relapses
Case 1	OU	Scotomas, photopsias OU, headache, back pain	17 yo, F	1. Quantiferon, serologies Lyme and syphilis, ACE, chest CT, brain MRI, and lumbosacral CT	20/25 OD; 20/60 (20/30 pH) OS	Yellow-white, gray placoid lesions in posterior pole, extending into the mid-periphery OU	Early hypofluorescence	Hypofluorescent in both eyes at early and late phase	HypoAF with hyperAF edges and late staining	EZ loss parafovea OU	M1, M3, M6, M14
Case 2	OD	Scotomas, photopsias OD, headache, neck pain, photophobia	15 yo, M	2. Negative HIV, Lyme, TB, toxacara, syphilis 3. Elevated CRP, +ve*T. gondii* (gM and IgG) 4. CXR – hilar fullness 5. Brain MRI, MRA, MRV unremarkable	20/400 OD; 20/20 OS	Bilateral yellow-white choroidal lesions scattered in posterior pole, extending to periphery OU, OD macular involvement	Early hypofluorescence, late hyperfluorescence	–	–	Thickened retina, disrupted foveal contour, focal subfoveal fluid, hyper-reflective lesions of outer retina layers	D2, W2
Case 3	OU	Acute central scotoma OS, with a generalized headache	22 yo, M	– Negative TPHA/VDRL, toxocariasis, leptospirosis, bartonella, tularemia, filariosis, CMV, HIV, Coxsackie virus, EBV enterovirus, and rubeola. 1. Negative ANA, C3/C4, anti-ECT, CRP, anti-DNA antibody, ESR, and CRP 2. Anterior chamber paracentesis negative for malignancy, negative PCR for HSV, CMV, VZV 3. EOG: Arden ratio: 210%/200% 4. mERG: macular dysfunction OS > OD 5. Brain MRI: stable hypersignal micronodular lesions of white matter	20/20 OD; 20/100 OS	+0.5 vitritis OS, focal RPE, EZ irregularities at macula	–	hypofluorescentfrom early to late phases	–	hyperreflective lesions of outer retina (RPE, EZ, ELM, ONL layers)	D10, M12, lost to follow-up, M36
Case 4	OU	Floaters, erythema, scotomas	24 yo, M	Negative quantiferon, syphilis, ACE, chest X-ray, brain MRI	20/20 OD, CF OS	Yellow-grey placoid lesions in posterior pole, extending into periphery OU, fovea involved OS, preretinal hemorrhage OD	Early hyperfluorescence, late hyperfluorescence/staining	–	Extensive HypoAF lesions with rare lesions with hyperAF edges a	Extensive EZ loss parafovea OD, foveal-involving OS	None

ACE, angiotensin converting enzyme; ANA, antinuclear antibody test; CBC, complete blood count; CF, counting fingers; CRP, C-reactive protein; CT, computed tomography; D, day; ESR, erythrocyte sedimentation rate; EZ, ellipsoid zone; FA, fluorescein angiography; FAF, fundus autofluorescence; ICG, indocyanine green angiography; M, month; RPE, retinal pigment epithelium; IS-OS, inner segment-outer segment; M, months; MRI, magnetic resonance imaging; OD, right eye; OS, left eye; OU, both eyes; SD-OCT, spectral-domain optical coherence tomography; TB, tuberculosis; W, week.

**Table 2:** Summary of treatments.

	Initial Tx	Tx at the first relapse	Tx at the second relapse	Tx at third relapse	Tx at 4threlapse
Relentless placoid chorioretinitis
Case 1	Prednisone = 1 mg/kg/day	MMF 250 mg BID + PO prednisone = 0.75 mg/kg/day	SQ adalimumab switched to IV tocilizumab	IV tocilizumab	–
Case 2	TMP-SMX = 320 mg BID IV MP500 mg/d for 2 days switched to PO prednisone 15 mg/day taper from 0.75 mg/kg/day	Adalimumab = 40 mg/2 weeks SQ + prednisone continued 10 mg/d PO	–	–	–
Case 3	IV MP 1,000 mg/3 days switched to prednisone PO taper (1 mg/kg/day)	IFN-alpha-2a for 3 MIU/daily switched to IV infliximab monthly	–	–	–
Case 4	Oral prednisone (1 mg/kg/day) for 22 weeks, then tapered down progressively over 10 weeks	–	–	–	–

Tx, treatments; IFN-alpha-2a, interferon-alpha-2a; MMF, mycofenolate mofetil; MP, methypprednisolone; TMP-SMX, trimethroprim-sulfamethoxazole; PO, oral administration; IV, intravenous; SQ, subcutaneous; BID, twice a day.

### Case 2: RPC

**Figure 4:**
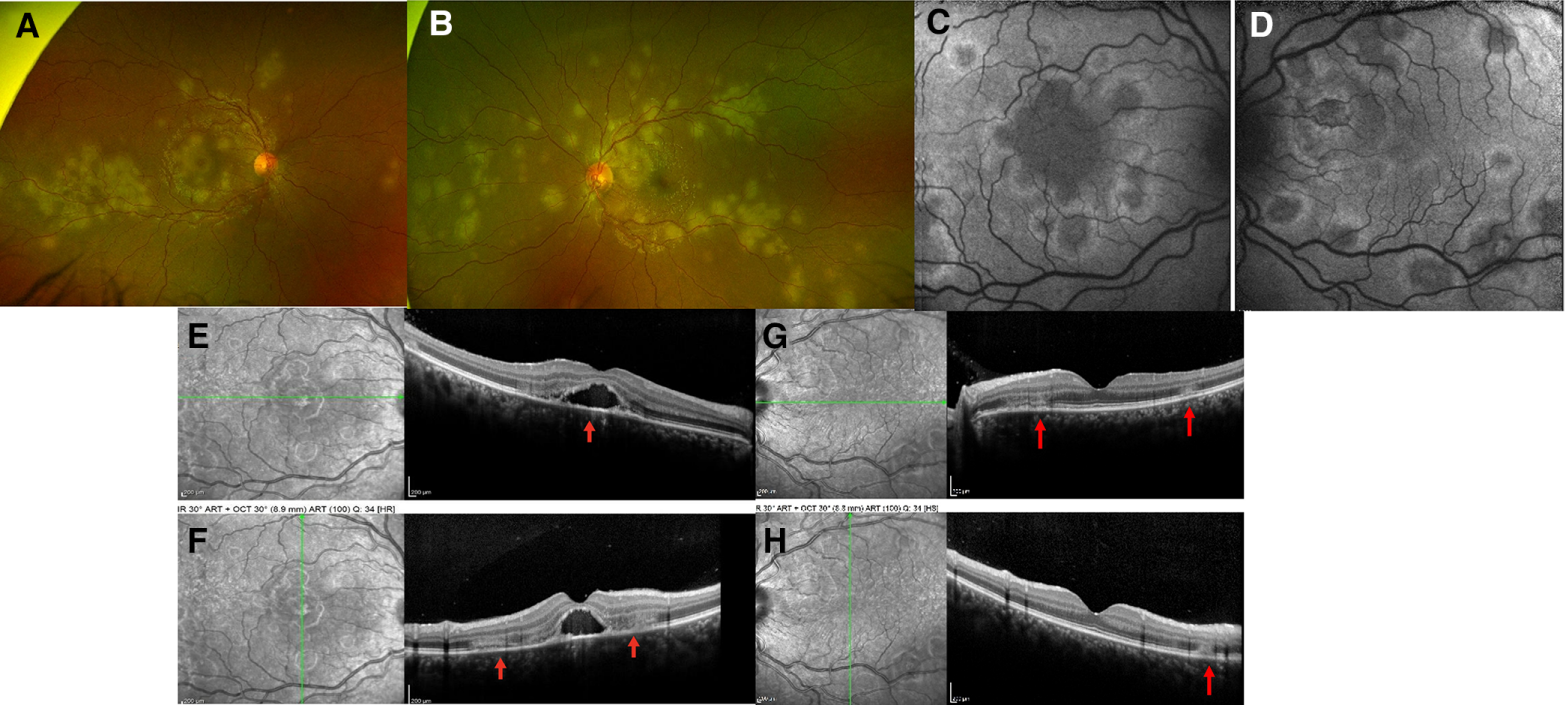
Patient 2. (**A,B**) Ultrawide-field fundus color imaging showed yellow-white placoid lesions in the posterior pole and extending into the mid-periphery. (**C,D**) Placoid lesions with central hypofluorescence and peripheral hyperfluorescence. (**E,F**) SD-OCT imaging demonstrated thickened retina, disrupted foveal contour, subfoveal and hyper-reflective lesions of the outer retina layers (red arrows) in both eyes. (**G,H**) After IV methylprednisolone, the SD-OCT demonstrated a resolution of subretinal fluid in the right eye, but the hyperreflective alterations persisted in the outer retina in the left eye and the EZ layer at the macula.

**Figure 5:**
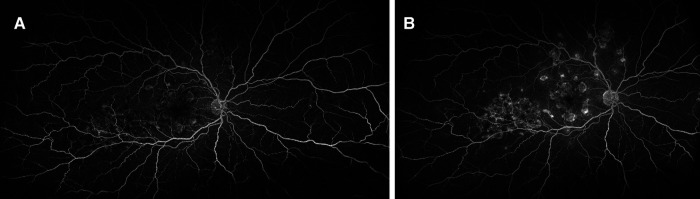
Patient 2. Fluorescein angiography: (**A**) early hypofluorescence with late (**B**) hyperfluorescence of the placoid lesions.

**Figure 6:**
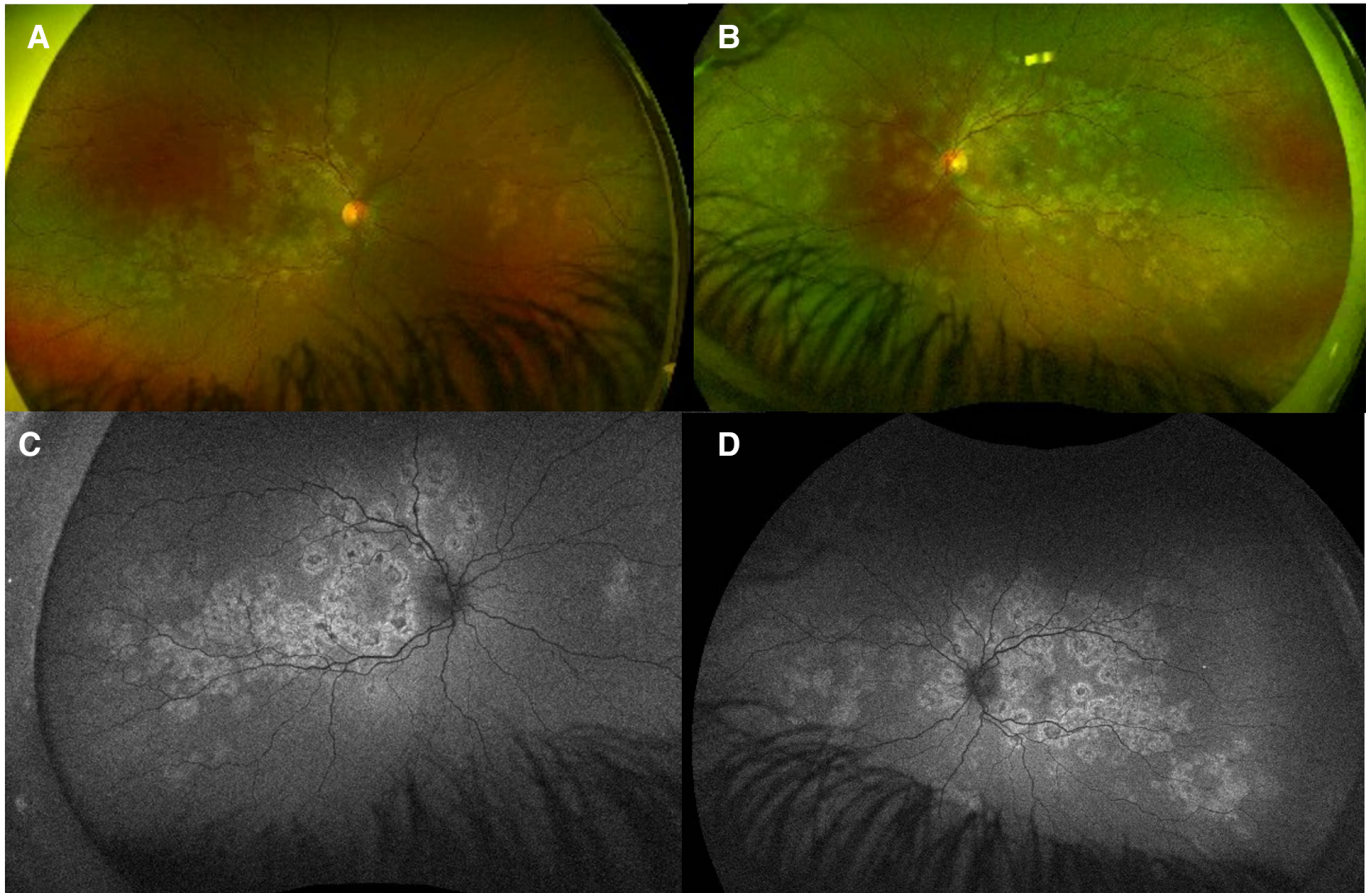
Patient 2. Eight months after the initial visit, on 40 mg/2 weeks adalimumab, visual acuity was drastically improved to 20/40 in the right eye eccentrically. (**A,B**) Ultrawide-field fundus color imaging showed more yellow-white placoid lesions in the posterior pole and extending into the mid-periphery compared to the initial presentation; (**C,D**) Placoid lesions with central hypofluorescence and peripheral hyperfluorescence.

A 15-year-old healthy boy who emigrated from Paraguay presented with scotomas and photopsias in his right eye for 1 week, along with headache, neck pain, and light sensitivity. He had increased blurry vision in the left eye for the past day. A review of symptoms was otherwise negative. His vision was 20/400 in the right eye and 20/20 in the left eye. The anterior chamber was quiet, but 1 + flare was noted in both eyes. Vitritis (1 + cells) was seen in the right eye. The fundus exam was significant for bilateral yellow-white choroidal lesions scattered in the posterior pole and extending to the periphery in both eyes with macular involvement in the right eye (Figures 4A,B). FAF imaging showed lesions with central hypoautofluorescence and peripheral hyperautofluorescence (Figures 4C,D). SD-OCT demonstrated thickened retina, disrupted foveal contour, focal subfoveal fluid, and hyper-reflective lesions of the outer retina layers in both eyes (Figures 4E,F). The patient was admitted to the hospital for a work-up and treatment. The uveitis work-up was positive for *Toxoplasma (T) gondii* IgM and IgG (Table 1). After consulting with the Infectious Disease Department, treatment for systemic toxoplasmosis infection was initiated with 6 weeks of oral trimethoprim-sulfamethoxazole and IV methylprednisolone because of the suspicion of APMPPE chorioretinal lesions (Table 2). After completion of 2 days of IV methylprednisolone, SD-OCT demonstrated a resolution of subretinal fluid in the right eye, but the hyperreflective alterations persisted in the outer retina in the left eye as well as those involving the EZ layer in the macula, bilaterally (Figures 4G,H). Oral FA showed early hypofluorescence with late hyperfluorescence of the fundus lesions indicative of active inflammation (Figures 5A,B). Wide-field color fundus pictures revealed progression since presentation with more widespread activity and new multifocal lesions in the mid-periphery with the healing of prior lesions. The patient was discharged 4 days after presentation with instructions to complete the 6-week trimethoprim-sulfamethoxazole course along with a PO prednisone 15-day taper. Five days after the initial presentation, the patient complained of subjective worsening of peripheral vision in his left eye and generalized headache. He was readmitted for 2 days of IV steroid infusions. FA showed diffuse healed placoid choroidal lesions and few active bilateral chorioretinal lesions. Two weeks after the initial presentation vision, the patient was on his last day of oral prednisone taper at 30 mg daily; SD-OCT showed bilateral retinal pigment epithelium (RPE) elevations with no further hyper-reflective outer nuclear layer (ONL) areas. Optos fundus pictures, however, showed additional active placoid subretinal lesions in the periphery of both eyes (Figures 6A,B) that appear hyperautofluorescent with a hypofluorescent center (Figures 6C,D). The patient was subsequently started on subcutaneous (SQ) adalimumab (40 mg/2weeks) and continued on 10 mg oral prednisone daily. One month after the initial presentation on 10 mg daily prednisone and adalimumab, the patient's right eye vision improved to 20/125 (from 20/400) and left eye vision remained stable at 20/20. Wide-field fundus pictures demonstrated continued healing of previously seen lesions without evidence of additional lesions. SD-OCT showed bilateral RPE elevations with no further hyperreflective areas in the ONL. After approximately 2 months of treatment with 10 mg prednisone daily and adalimumab every 2 weeks, the patient was seen for retinal imaging testing and telemedicine visit and subjectively reported improved vision back to baseline in both eyes. Wide-field fundus color imaging showed healing lesions in the periphery of both eyes with no new lesions. Five months following the initial presentation, SD-OCT imaging showed fewer subretinal deposits at the fovea of the right eye compared to 3 months prior and unchanged macular contour and outer retina of the left eye. He remained stable on adalimumab with inactive lesions on multimodal testing (fundus photos, FAF, and OCT) at 9 months from the presentation.

### Case 3: RPC

**Figure 7:**
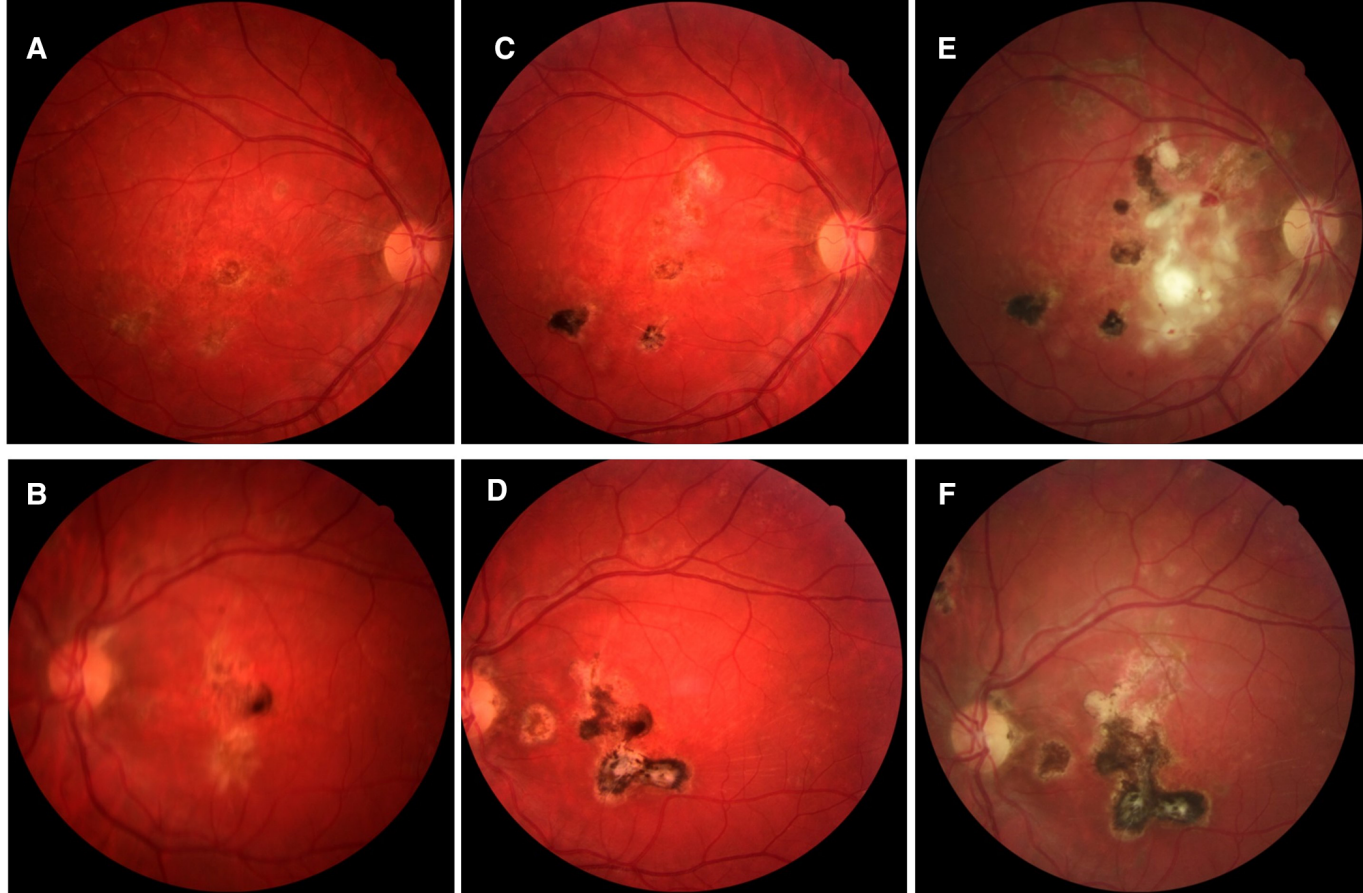
Patient 3. (**A,B**) Fundus pictures showing posterior retinal lesions occurring simultaneously with macular involvement. (**C–F**) At 12 and 36 months, the lesions look older, healing pigmented but more numerous and widespread.

**Figure 8:**
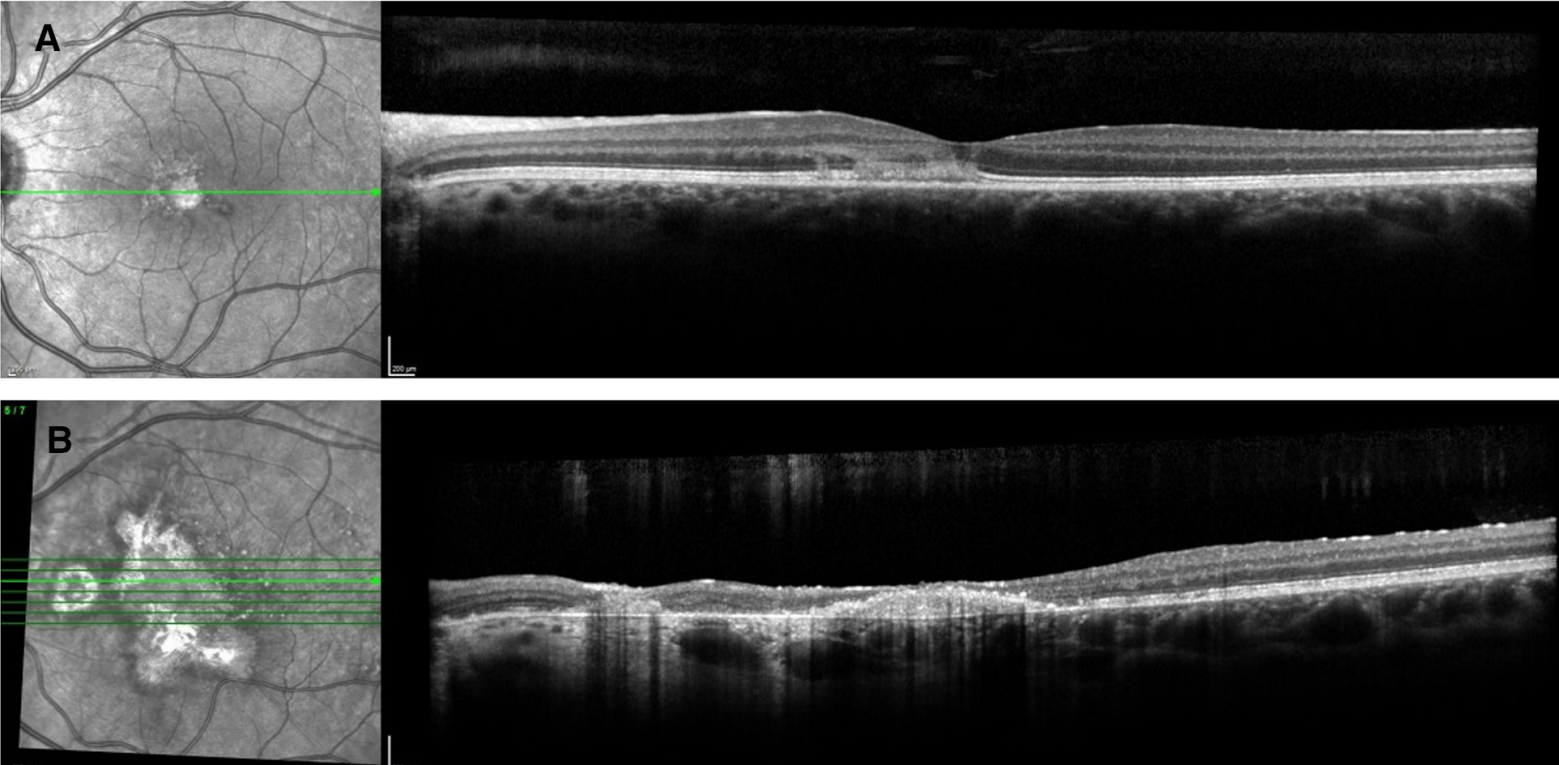
Patient 3. (**A**) At presentation, spectral-domain and optical coherence tomography angiography (SD-OCT) showed right hyperreflective lesions of the outer retinal involving the retinal pigment epithelium, the ellipsoid zone, and outer nuclear layers. (**B**) At 12 months, progression of the macular retinal pigmentary inflammatory lesions with inflammation of the outer retinal and sub- and intraretinal deposits and focal photoreceptor/RPE atrophy.

**Figure 9:**
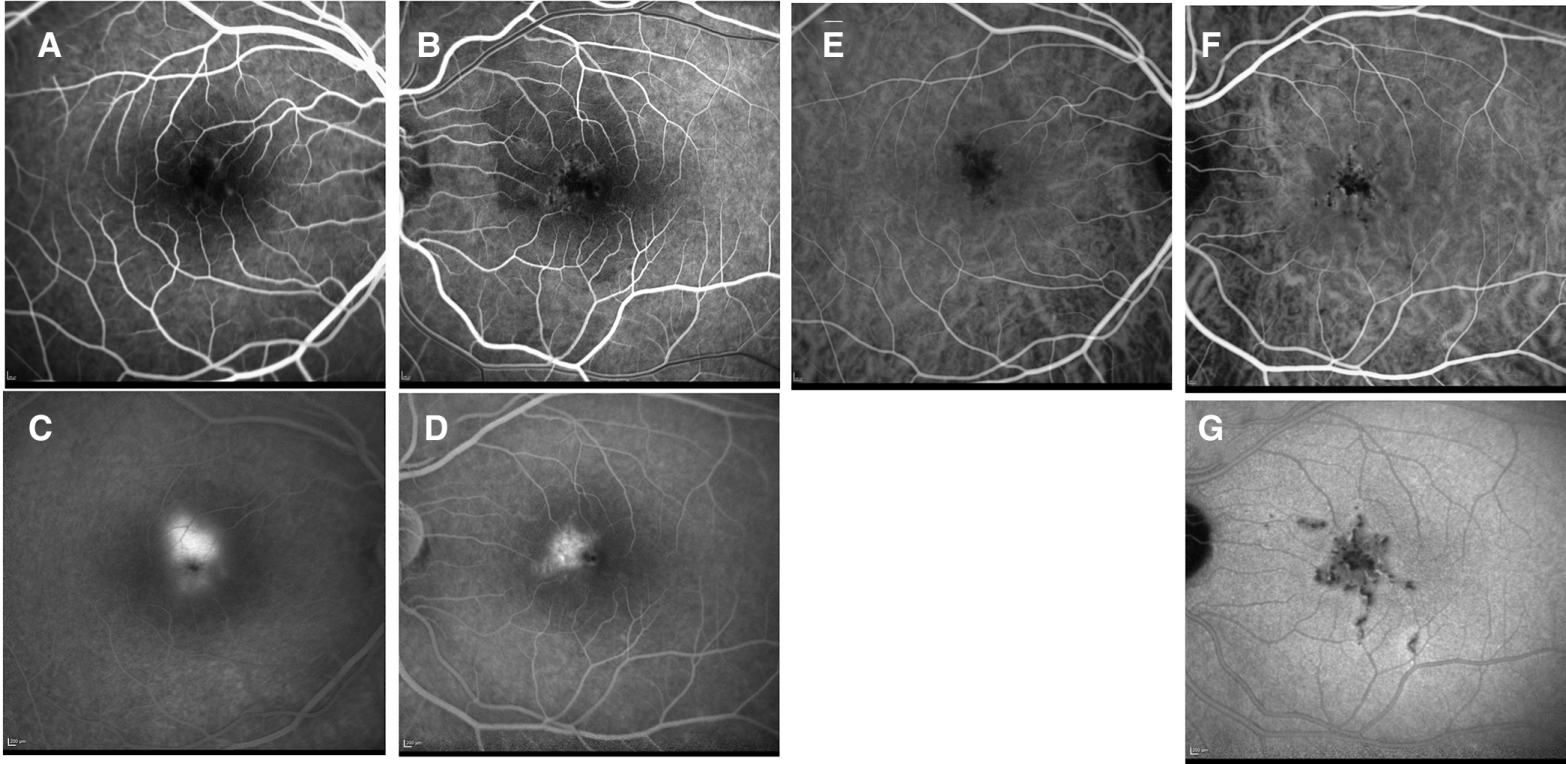
Patient 3. (**A,B**) Lesions were hypofluorescent at the early phase of FA, (**C,D**) with late leakage, and (**E–G**) Hypofluorescent on all phases of ICG.

**Figure 10:**
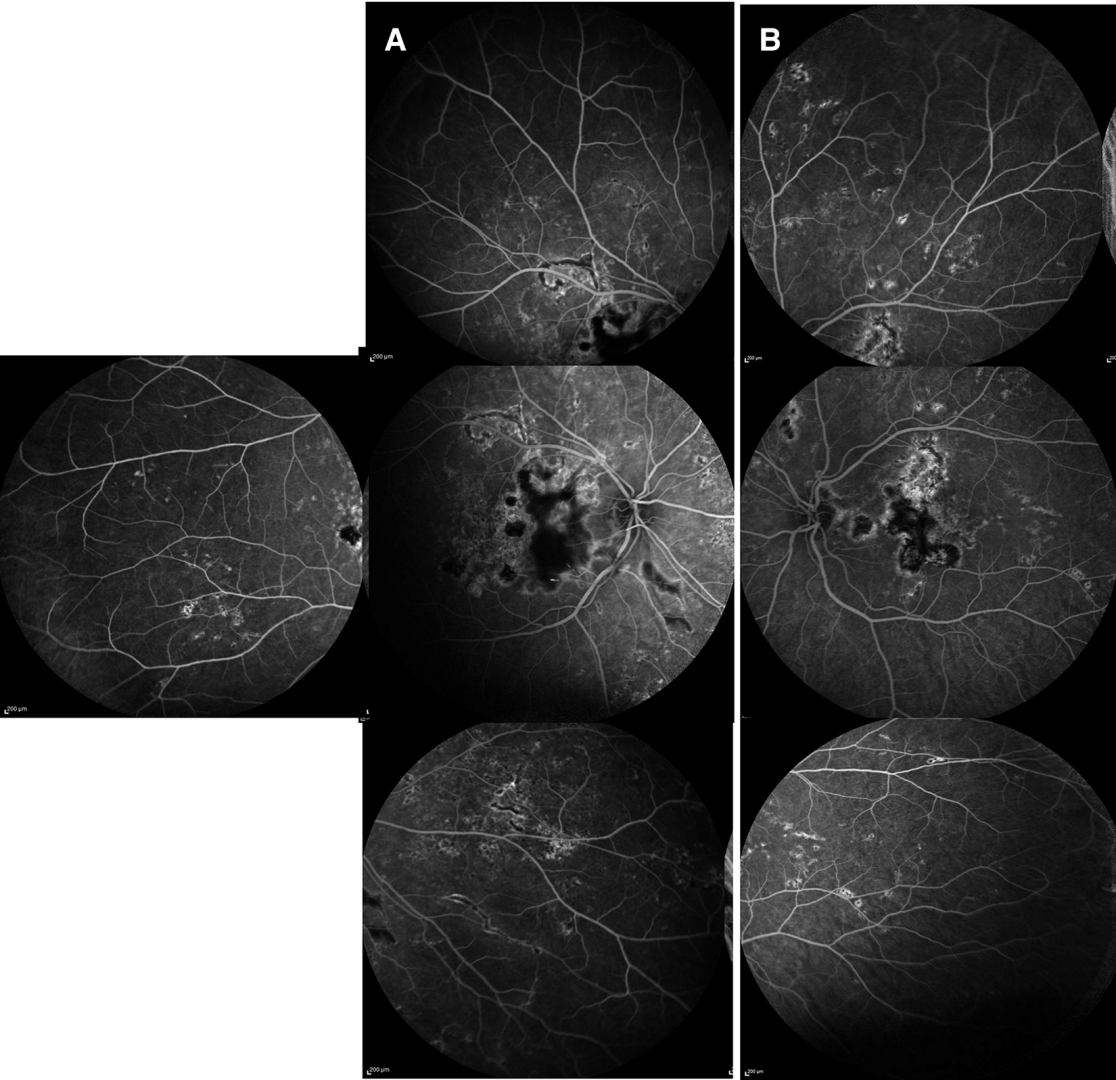
Patient 3. (**A,B**) At last follow-up, intermediate phase of fluorescein angiography (FA) of both eyes showing hypofluorescence of the lesions in the posterior pole with hyperfluorescent borders and extensive widespread new lesions with staining.

A 22-year-old previously healthy men working in a pet store presented to the emergency department for an acute central scotoma in the left eye with a generalized headache. The visual acuity was 20/20 in the right eye and 20/100 in the left eye, with a left relative afferent pupillary defect. The fundus exam showed +0.5 vitritis in the left eye associated with focal irregularities in the RPE and EZ in the macula on SD-OCT.  The work-up was negative, including anterior chamber paracentesis that ruled out malignant cells, and HSV, CMV, and VZV PCR were negative. After ruling out intestinal parasites by the fecal test, the patient was given IV methylprednisolone, with a subsequent oral (PO) steroid taper starting at 1 mg/kg/day (doses details are in Table 2). Ten days after the initial presentation, a new activity was detected in the fellow eye in the form of a macular chorioretinal lesion (Figure 7A). SD-OCT showed right hyper-reflective lesions of the outer retinal involving the RPE, EZ, external limiting membrane (ELM), and ONL layers and new hyperreflective lesions on SD-OCT at the macula in the left eye (Figure 8A). The lesions were hypofluorescent in the early phase of FA (Figures 9A,B) with late hyperfluorescence (Figures 9C,D) and hypofluorescent in the early to late phases of ICG (Figures 9E–G). The patient was transitioned from oral prednisone (0.75 mg/kg/d) to interferon-alpha-2a (IFN alpha-2a) (Table 2). His visual acuity improved from 20/200 to 20/100 in the right eye but remained at hand motion (HM) in the left eye. The size of the outer retinal lesions still increased but at a slower rate than when on oral steroids alone. The patient suffered from major depressive disorder along with suicidal attempts and therefore was started on monthly IV infusions of infliximab. Unfortunately, due to multiple missed appointments and treatments, when the patient was reviewed 12 months after the initial presentation, the chorioretinal lesions had progressed within the posterior pole of both eyes (Figures 7C,D). On SD-OCT, the lesions were hyper-reflective from occupying space from the choroid to the outer retina through the inner retina and associated with thinning of the retina (Figure 8B). Infliximab was restarted. An electrophysiology study was conducted 1 year after the presentation to rule out inherited diseases, bestrophinopathies, and paraneoplastic retinopathies. Findings included a normal electroretinogram, an electrooculogram Arden ratio of 210%/200%, and a multifocal electroretinogram significant for macular dysfunction in the left eye greater than the right eye. The visual evoked potential worsened compared to the initial presentation. The work-up was completed by repeated brain MRI that revealed stable hypersignal micronodular lesions of the white matter. The patient was lost to follow-up for another year and was reviewed for recent peripheral vision loss. He had stopped the treatment with infliximab. His visual acuity was stable at worse than 20/200 in both eyes, but new lesions continued to develop in the mid-periphery of both eyes (Figures 7E,F). The patient was restarted on monthly infliximab infusions, and due to the continued progression of lesions 3 years after the initial presentation (Figure 10), the patient was switched to adalimumab (40 mg/1 week), which finally stopped additional new lesions.

### Case 4: RPC

A 24-year-old men with no past ocular/medical history presented with a 2-month history of floaters, erythema and scotomas bilaterally. He was initially seen by an outside ophthalmologist who noted bilateral diffuse placoid lesions, neovascularization, and vitreous hemorrhage in the right eye, for which he received an intravitreal bevacizumab injection. He was then referred to a university uveitis service.

**Figure 11:**
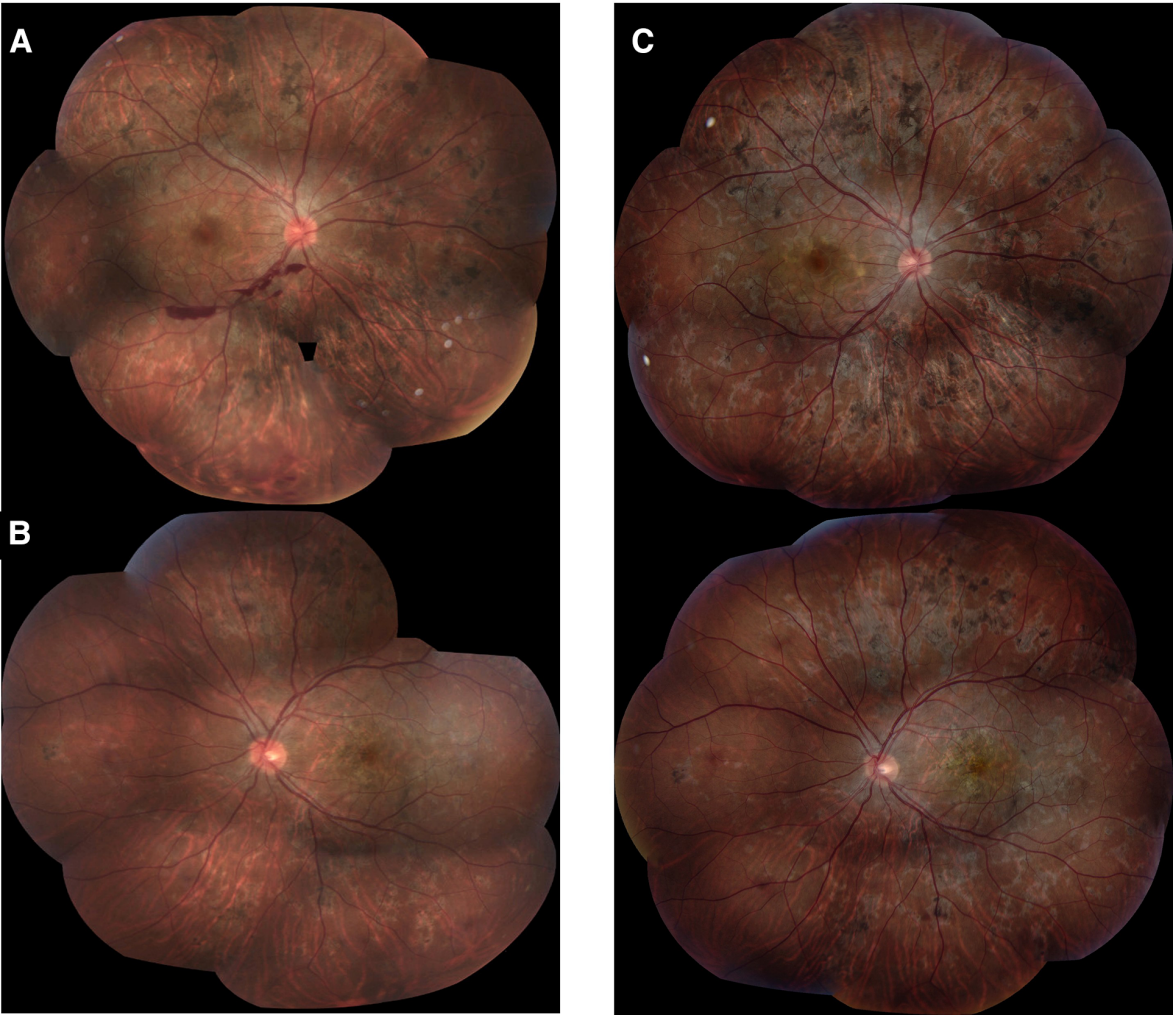
Patient 4. (**A,B**) At presentation, fundus photographs of both eyes showing extensive white/grey placoid lesions in the posterior pole and extending into the periphery, with preretinal heme OD. Lesions appear mostly atrophic and inactive. (**B,C**) At 5 month follow up, there appears to be no obvious progression of disease or active lesions.

**Figure 12:**
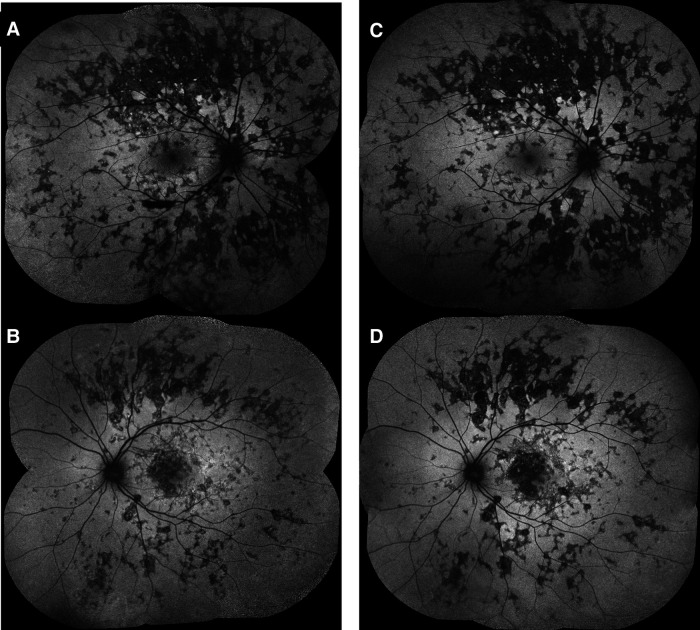
Patient 4. (**A,B**) At presentation, fundus autofluorescence (FAF) of both eyes showing extensive hypofluorescent lesions with very few scattered lesions with hyperfluorescent edges. (**C,D**) At 5 month follow up, there appears to be no obvious new lesions/progression or active hyperfluorescent lesions.

**Figure 13:**
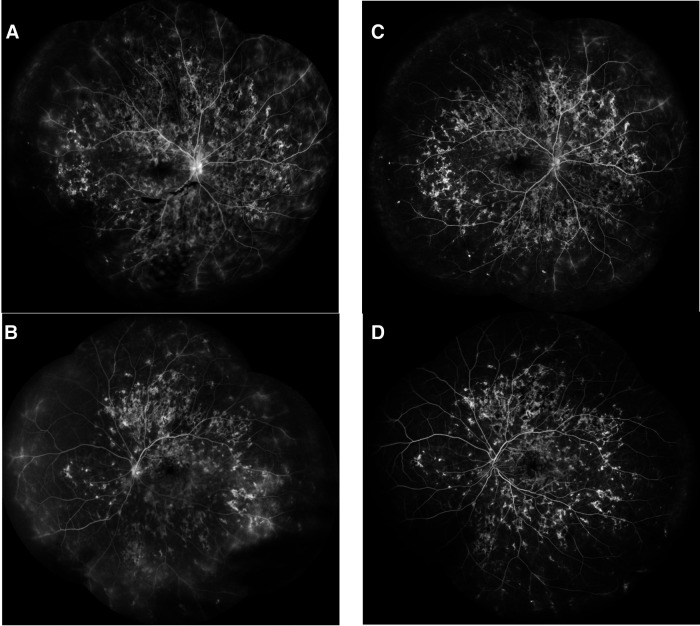
Patient 4. (**A,B**) At presentation, late phase fluorescein angiography (FA) of both eyes showing diffuse late staining of the lesions, with extensive small vessel vasculitis/ferning. There is no neovascularisation of the disc or retina. (**C,D**) At 5 month follow up, there was regression of the mid-peripheral and peripheral small vessel vasculitis, with no evidence of recurrence of neovascularisation of the disc.

On the initial exam, his vision was 20/20 in the right eye and counting fingers in the left eye. A slit lamp exam showed 1 + cells and flare in the right eye and 3 + cells/2 + flare in the left eye. There were 0.5 +  anterior vitreous cells in the right eye and 1 + anterior vitreous cells in the left eye. Gonioscopy showed bilaterally open angles, with white inflammatory material in the angles at 3 and 8 o'clock in the left eye. The fundus examination revealed yellow and gray placoid lesions (of varying stages/pigmentation) in the posterior pole and extending into the mid-periphery in both eyes, as well as preretinal hemorrhage in the right eye (Figures 11A,B). FAF showed extensive hypofluorescent lesions with very few scattered lesions with hyperfluorescent edges (Figures 12A,B). On FA, both eyes demonstrated diffuse late staining of the lesions, with extensive small vessel vasculitis/ferning and regression of the disc neovascularization previously noted in the right eye (Figures 13A,B). SD-OCT revealed extensive EZ layer and outer retinal loss in the parafovea of the right eye and extensive EZ/outer retinal loss involving the fovea in the left eye (Figures 13AB).

**Figure 14:**
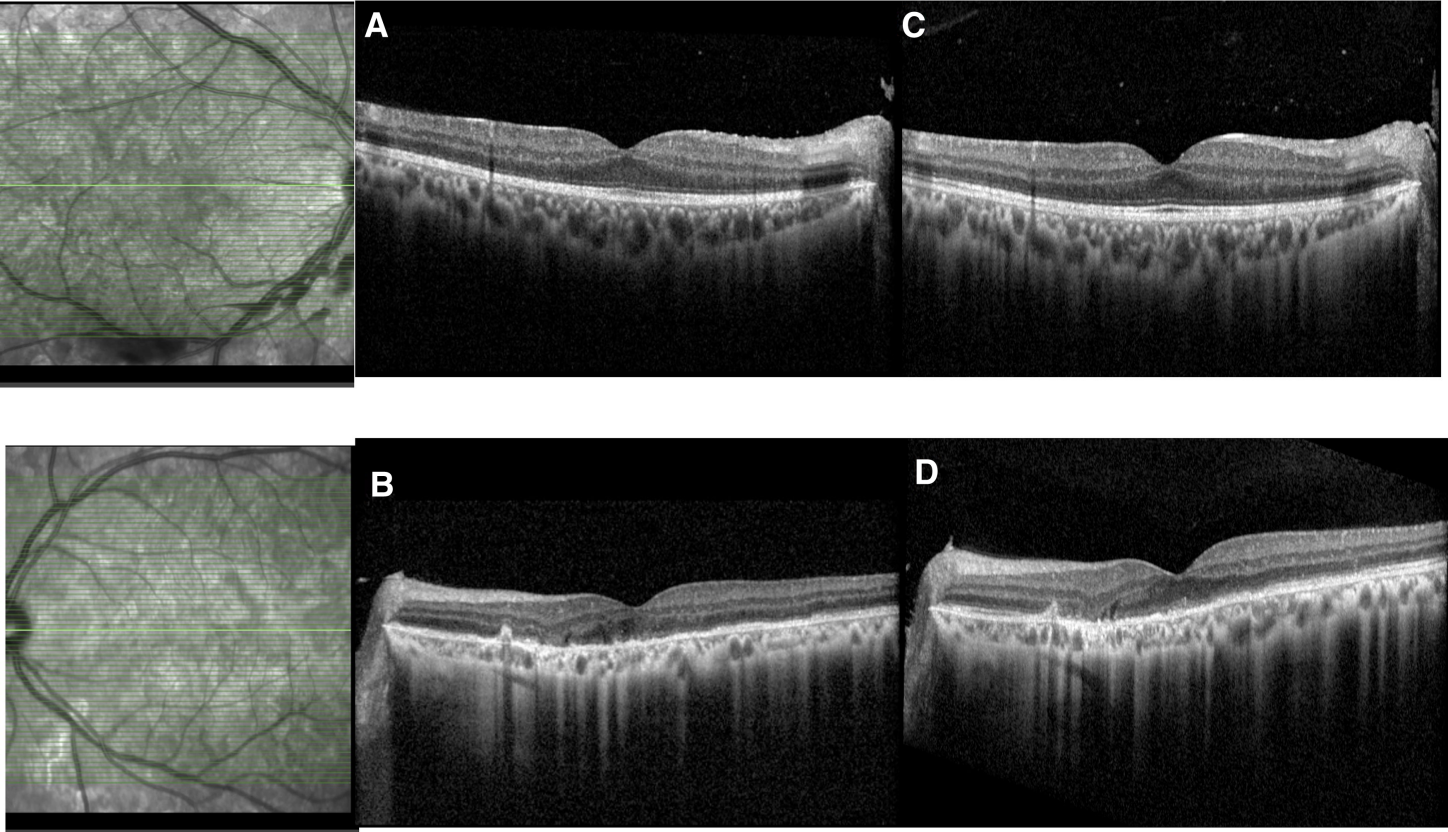


A complete uveitis work-up was negative (Table 1). The patient was started on oral prednisone 60 mg and topical prednisolone acetate drops 6×/day in both eyes. On follow-up visit 1 month after the initial presentation, the vision was stable at 20/20 in the right eye and had significantly improved to 20/70 (from counting fingers) in the left eye. Wide fields fundus pictures and FAF demonstrated no new lesions or progression/activity of previously seen lesions. Oral prednisone was slowly tapered over the course of 3 months. At 5 months of follow-up, the vision had improved to 20/60 in the left eye. There was no evidence of disease recurrence or progression on fundus pictures (Figures 11C,D) or FAF (Figures 12C,D) off oral prednisone. FA showed regression of small vessel vasculitis and no evidence of active lesions (Figures 13C,D). SD-OCT showed stable EZ loss with no significant recovery, despite significant improvement in vision (Figures 14C,D). The improvement has been maintained for over 1 year, off therapy. Immunomodulatory therapy was not initiated, as the disease did not recur after steroid therapy.

## Discussion

Due to the recurrence of bilateral lesions over 6–36 months (two patients) without or despite a steroid-sparing therapy, and/or because of the atypical location of the multiple lesions (>50) extending from the posterior pole to the equator and mid-peripheral retina (all four patients), we diagnosed these four patients as RPC to differentiate them from other commonly associated white dot syndromes, such as APMPPE and SC. This contrasts with APMPPE, which presents chorioretinitis lesions limited to the posterior pole. The lesions are smaller than those in APMPEE (<1/2 disc area) and are classically bilateral. In both entities, fresh lesions may develop, so lesions of different ages can be seen. Additionally, anterior uveitis and mild vitritis may be seen in both diseases. The duration of the disease and the persistent course were the key differences from APMPPE and SC. It is important that RPC is diagnosed early due to the increased threat of vision loss; however, even if diagnosed early, as represented in our four cases, RPC can have a poor visual recovery if an effective treatment has not been initiated. Guidelines for standard treatment for RPC still do not exist. Thus, this paper serves as an analysis of our cases and previously published cases of RPC as a means to contribute to the current knowledge and treatment options for RPC.

Clinically, two of our cases had initial symptoms of photopsias, scotomas, and persisting headaches. All four patients were initially started on high-dose oral or IV steroids and later transitioned to additional immunosuppressive agents or biologics for three of them and steroid tapering for all of them. The first case describes the use of oral prednisone, the inefficacy of conventional immunosuppressive therapy, MMF, and finally the efficacy of biologics, adalimumab, tocilizumab, and infliximab. The second case describes the use of IV solumedrol, oral prednisone, and the efficacy of SQ/2 weeks adalimumab started as early as during the first month of RPC presentation. The third case treatments were IV methylprednisolone, failure of IFN-alpha-2a, and finally the efficacy of IV infliximab with SQ/1 week adalimumab when the patient finally showed compliance to anti-TNF-alpha treatments. The fourth case received only oral and topical steroids with slow tapering over a course of 3 months without relapse.

Quiescence of the chorioretinitis lesions was obtained after 7 months (patient number 1), 1 month (patient numbers 2 and 4), and 36 months (patient number 3) but with problems of treatment compliance in patient number 3. Despite the response to treatment, the final visual acuity was affected in all four patients.

As regards previously published treatments for RPC (Table 3), systemic steroids are typically used as first-line treatments, as they assist in the healing of lesions and slowing progression. Despite this, combination immunosuppressive therapy is almost universally needed for the prevention of relapses, disease control, and systemic steroid tapering. Jones et al. (1) described the use of prednisone alone in one case where visual acuity remained impaired after cessation and more than 100 pigmented lesions were present 28 months after initial presentation. Similarly, Khalifa et al. (18) reported the use of oral prednisone (1 mg/kg/day) alone with successful tapering within 3 months without recurrence; however, due to extensive macular scarring, final visual acuity was impaired. This suggests that although the use of prednisone alone may prevent the initial recurrence of lesions, impaired visual acuity and future recurrences are still probable. Other immunosuppressants (i.e., cyclosporine and azathioprine) are typically added in combination with prednisolone due to relapse. We describe a different therapeutic approach for every patient based on ophthalmologists/rheumatologists and internal medicine preferences in our four different centers.

**Table 3:** Summary of previously reported cases of RPC in the literature.

Authors	Treatments with doses	Timing of quiescence from initial presentation/initiation of treatment	Duration of follow-ups with quiescence	Findings/recommendations
Khalifa et al. (18)	Oral prednisone (1 mg/kg/day)	3 months	– (not specified)	1. Visual prognosis is poor in RPC 2. Despite treatment, extensive macular scarring remained with reduced final VA after quiescence 3. There may be relationship between quadrivalent HPV vaccination and immunologic triggers leading to posterior non-infectious uveitis
Uraki et al. (15)	Case 1: methylprednisolone 1,000 mg/day × 3 days, followed by prednisone 60 mg/day with tapering, 40 mg STTA, cyclosporine 250 mg/day Case 2: methylprednisolone 1,000 mg/day × 3 days, 60 mg/day with tapering, 40 mg STTA, then cyclosporine 200 mg/day and 40 mg/day prednisone Case 3: 40 mg of STTA and prednisone 15 mg/day, then cyclosporine 250 mg/day + 30 mg/day Case 4: Prednisone 60 mg/day with tapering, then cyclosporine 170 mg/day with prednisone 20 mg/day	Case 1: not specified Case 2: recurrences for 8 months Case 3: 8 months Case 4: not specified	Case 1: no severe recurrences for 48 months but occasionnally recurrences with peripheral retinal exudates Case 2: 42 months Case 3: not specified Case 4: 48 months	1. Cyclosporine and prednisone combination therapy is considered a potential therapeutic strategy for RPC
Jones et al. (1)	Case 1: – Topical steroids + acyclovir PO 800 mg × 5 times/day – then, IV foscarnet, then IV ganciclovir – then, prednisone PO 50 mg/d + famciclovir 500 mg PO, TID – then, cyclosporine PO 100 mg/d Case 2: – prednisone PO 60 mg/d – periocular steroid injection OD Case 3: – prednisone PO 80 mg/d with taper – then, add cyclosporine PO 150 mg/d Case 4: – no treatment Case 5: – prednisone PO (80 mg/day), doxycycline (100 mg BID) Case 6: – prednisone PO (50 mg/day)	Case 1: continued recurrence of lesions at 30 months after initial presentation. Case 2: continued recurrence of lesions at 22 months after initial presentation Case 3: limited progression of lesions following initiation of prednisone 6 months after initial presentation.	Case 1: not specified Case 2: not specified Case 3: 15 months Case 4: 11 months Case 5: 13 months Case 6: 28 months	1. The use of systemic corticosteroids lead to healing of chorioretinal lesions and improvement of visual acuity from initial presentation 2. Suggests that the disease can still progress despite steroids therapy 3. More research must be done to determine the role of corticosteroids, antiviral medications, and cyclosporine
Yeh et al. (8)	Prednisone PO (20 mg/day); MMF 1,000–2,000 mg/day	2 months approximately 5 months after initial presentation	18 months	1. Support the use of conventional immunosuppressive therapy for RCP
Amer et al. (19)	IV MP (500 mg/day × 3 days); prednisolone taper (1 mg/kg/day); then, MMF (500 mg × 2/day) for 1 month	approximately 4 months after initial presentation	15 months	OCT imaging is valuable tool for assessment of the protean morphologic changes.
Roth et al. (17)	IVTA (4 mg) biannually for 3 years	not specified.	63 months after initial presentation	
Jyotirmay et al. (20)	– Prednisolone + azathioprine (13 patients) – Prednisolone + azathioprine + cyclosporine (1 patient) – Azathioprine (2 patients) – Prednisolone + azathioprine + PSTTA (1 patient) – Steroids + azathioprine + IV MP (7 patients)	The shortest time to recurrence was 6 months and the longest was after 5 years.	not specified	1. Most controlled with prednisolone combined with a single immunosuppressive agent azathioprine. Occasionally a third immunosuppressant (cyclosporine), pulsed methyl prednisolone (in case of macula involvement), or PSTK inj. were required to control relapses or recurrence. Recommend PSTK inj.
Asano et al. (16)	Case 1 – Prednisolone PO (15 mg/day), cyclosporine (200 mg/day) +adalimumab (40 mg/2 weeks) Case 2 – Prednisolone PO (15 mg/day) +cyclosporine (150 mg/day) followed by termination of cyclosporine + initiation of adalimumab (40 mg/2 weeks)	Case 1: At 17 months after presentation initiation of adalimumab because of recurrence. 3 months after adalimumab, cyclosporine was terminated without recurrence. Case 2: At 9 months after initiation of adalimumab.	Case 1: 1 year after initiation of adalimumab, oral prednisolone was successfully terminated without recurrence Case 2: 9 months after initiation of adalimumab, inactivation of inflammation was detected	1. After adalimumab was used for treatment of RPC, corticosteroids were able to be tapered and no relapses were seen 2. The use of adalimumab for RPC may have potential to effectively suppress recurrence of lesions

MP, methylprednisolone; STTA, subtenon triamcinolone acetate injection.

Uraki et al. (15) found that in their four reported cases of RPC, a combination of prednisolone 25–30 mg/day and cyclosporine 170–250 mg/day along with subtenon triamcinolone acetate injection in two of the patients resulted in no recurrence of lesions across each of the four cases for 8–48 months. Jones et al. (1) presented two cases where cyclosporine (100–150 mg/day) was added in combination with prednisolone with successful tapering of prednisolone without any recurrence of lesions. Two additional studies have found the effective use of prednisolone and MMF 1,000–2,000 mg/day with no recurrence of lesions for 15–18 months: Yeh et al. (8) reported that remission was achieved following 2 months of MMF treatment with successful tapering of oral prednisone (initial dose 20 mg/day), while Amer et al. (19) reported a case where a 3-day course of IV methylprednisolone (500 mg/day) with a tapering dose of prednisolone (1 mg/kg/day) and addition of MMF (500 mg × 2/day) for 1 month lead to no recurrence of lesions 15 months after the first presentation.

Despite the effectiveness of immunosuppressive agents, treatment can be further complicated in patients with risk factors for medication use. Roth et al. (17) reported a pregnant woman with RPC who was treated with intravitreal triamcinolone (IVTA) due to the risk of teratogenic effects of immunosuppressive agents. The patient was treated with 4 mg IVTA biannually for 3 years with preservation of vision at 20/25 at the 63-month follow-up from the initial presentation. IVTA improved the chorioretinitis lesions and visual acuity in this case; however, the patient developed a posterior subcapsular cataract and noninfectious endophthalmitis, likely caused by its use. Jyotirmay et al. (20) presented 26 eyes of 16 patients diagnosed with RPC. Of these, 13 patients were maintained on oral prednisolone (1.5 mg/kg/day) and azathioprine (1.5–2.0 mg/kg/day) and tapered until a minimum dose was reached without recurrence of lesions. In seven patients, 3 days of IV methylprednisolone pulse therapy (1 g/day) was given, followed by oral steroids and azathioprine due to macular involvement. Of the other cases, two eyes were treated with azathioprine alone, one eye was treated with prednisolone and posterior subtenon triamcinolone injection (STTA), and one eye was treated with steroid, azathioprine, and cyclosporine combination. Again, all of these cases reached a point of quiescence. Of all the cases presented in this report, 96.2% had favorable visual outcomes, with two patients showing no improvement in visual acuity despite IV methylprednisolone and maintained on oral steroids and azathioprine. Asano et al. (16) in a case series of two patients with RPC found effectiveness of the combination of prednisolone, cyclosporine, and adalimumab. In their first case, they present the use of oral prednisolone (15 mg/day), cyclosporine (200 mg/day), and adalimumab (40 mg/2 weeks) with successful tapering of corticosteroids after 1 year and immunomodulatory treatments after 3 months without any adverse effects or recurrence of lesions. In the second case, oral prednisolone (15 mg/day) and cyclosporine (150 mg/day) were initiated due to the recurrence of lesions, and after ocular inflammatory changes subsided, cyclosporine was subsequently terminated and adalimumab (40 mg/2 weeks) was started. Both cases reported by Asano et al. (16) are still treated with adalimumab at the time of the final visit; thus, the optimal duration of adalimumab treatment and tapering has yet to be determined.

Each of the four cases presented herein was treated with a combination of prednisolone and an anti-TNF or anti-IL-6 medication (i.e., adalimumab, infliximab, or tocilizumab). Two cases displayed a failure of MMF and interferon-alpha-2a in preventing flares of RPC. Other cases published in the literature have used adalimumab effectively, as discussed above; however, there have been no reports of treatment with infliximab. Previous papers have shown the efficacy of treatment with systemic steroids with slow tapering, with intravitreal steroids, and with subtenon steroid injections (Table 3). On the contrary, in our cases of RPC, we observed relapses when tapering oral steroids in three of the four cases but efficacy at obtaining the eye inflammation quiescence with slow steroid tapering over a course of 3 months in one patient. Due to the sight-threatening and chronic course of RPC, several reports have described the use of steroid-sparing agents to treat RPC. Several have also used efficient oral cyclosporine and oral azathioprine or MMF (Table 3). The first case here was unsuccessfully treated with MMF, and upon literature review, we have not encountered another report of IFN-alpha-2a being successfully used for RPC.

To our knowledge, this is the first study to present a case of RPC that responded to an anti-IL-6 agent (i.e., tocilizumab) for treating RPC. Our findings suggest that biologics, like anti-TNF-alpha agents (i.e., adalimumab and infliximab) and IL-6 agents, such as tocilizumab, may effectively prevent recurrences of RPC; however, MMF and IFN-alpha-2a should perhaps be avoided. However, we acknowledge the limitations of our study, given the small number of cases presented due to the rarity of RPC.

## Conclusion

Here, we reviewed the diagnostic approach to early identification and treatment of RPC by distinguishing it from the similar counterpart APMPPE, which classically does not require treatment. Furthermore, we review treatment strategies for RPC and their anecdotal success in this case series of four patients and results from the current literature. This is the first study, to our knowledge, to report a case with the successful use of an anti-IL-6 agent (i.e., tocilizumab) for RPC. In our case series and upon literature review, we found that biologics like anti-TNF-alpha (adalimumab and infliximab) and anti-IL-6 (tocilizumab) may be more effective at RPC control, and MMF and IFN-alpha-2a may be insufficient to control RPC flares.

## Data Availability

The original contributions presented in the study are included in the article/Supplementary Material; further inquiries can be directed to the corresponding author.
